# Tracking of Chromosome and Replisome Dynamics in *Myxococcus xanthus* Reveals a Novel Chromosome Arrangement

**DOI:** 10.1371/journal.pgen.1003802

**Published:** 2013-09-19

**Authors:** Andrea Harms, Anke Treuner-Lange, Dominik Schumacher, Lotte Søgaard-Andersen

**Affiliations:** Department of Ecophysiology, Max Planck Institute for Terrestrial Microbiology, Marburg, Germany; Institute of Molecular and Cell Biology (IMCB), A*STAR, Singapore

## Abstract

Cells closely coordinate cell division with chromosome replication and segregation; however, the mechanisms responsible for this coordination still remain largely unknown. Here, we analyzed the spatial arrangement and temporal dynamics of the 9.1 Mb circular chromosome in the rod-shaped cells of *Myxococcus xanthus*. For chromosome segregation, *M. xanthus* uses a *parABS* system, which is essential, and lack of ParB results in chromosome segregation defects as well as cell divisions over nucleoids and the formation of anucleate cells. From the determination of the dynamic subcellular location of six genetic loci, we conclude that in newborn cells *ori*, as monitored following the ParB/*parS* complex, and *ter* regions are localized in the subpolar regions of the old and new cell pole, respectively and each separated from the nearest pole by approximately 1 µm. The bulk of the chromosome is arranged between the two subpolar regions, thus leaving the two large subpolar regions devoid of DNA. Upon replication, one *ori* region remains in the original subpolar region while the second copy segregates unidirectionally to the opposite subpolar region followed by the rest of the chromosome. In parallel, the *ter* region of the mother chromosome relocates, most likely passively, to midcell, where it is replicated. Consequently, after completion of replication and segregation, the two chromosomes show an *ori*-*ter*-*ter*-*ori* arrangement with mirror symmetry about a transverse axis at midcell. Upon completion of segregation of the ParB/*parS* complex, ParA localizes in large patches in the DNA-free subpolar regions. Using an Ssb-YFP fusion as a proxy for replisome localization, we observed that the two replisomes track independently of each other from a subpolar region towards *ter*. We conclude that *M. xanthus* chromosome arrangement and dynamics combine features from previously described systems with new features leading to a novel spatiotemporal arrangement pattern.

## Author Summary

Work on several model organisms has revealed that bacterial chromosomes are spatially highly arranged throughout the cell cycle in a dynamic yet reproducible manner. These analyses have also demonstrated significant differences between chromosome arrangements and dynamics in different bacterial species. Here, we show that the *Myxococcus xanthus* genome is arranged about a longitudinal axis with *ori* in a subpolar region and *ter* in the opposite subpolar region. Upon replication, one *ori* remains at the original subpolar region while the second copy in a directed and *parABS*-dependent manner segregates to the opposite subpolar region followed by the rest of the chromosome. In parallel, *ter* relocates from a subpolar region to midcell. Replication involves replisomes that track independently of each other from the *ori*-containing subpolar region towards *ter*. Moreover, we find that the *parABS* system is essential in *M. xanthus* and ParB depletion not only results in chromosome segregation defects but also in cell division defects with cell divisions occurring over nucleoids. In *M. xanthus* the dynamics of chromosome replication and segregation combine features from previously described systems leading to a novel spatiotemporal arrangement pattern.

## Introduction

In all cells, chromosome replication and segregation are closely coordinated with cell division to ensure that daughter cells inherit the correct chromosome complement. In eukaryotic cells, replication and chromosome segregation are temporally separated and segregation is accomplished by a highly conserved mitotic machinery [1]. In contrast, chromosome replication and segregation occur concomitantly in bacteria [2]. Moreover, bacterial chromosomes are spatially precisely arranged with individual chromosomal loci reproducibly localizing to the same subcellular locations [3]–[9] and this spatial arrangement is established during the segregation process [3], [4], [7], [10]. The mechanistic details underlying the linked processes of chromosome segregation and spatial arrangement in bacteria are much less understood than in the case of their eukaryotic counterparts and have only been studied in details in a few species. Interestingly, these analyses have revealed significant differences in chromosome arrangement as well as in the mechanisms of chromosome segregation in different bacterial species.

Bacteria typically contain a single circular chromosome. Replication initiates at a well-defined origin of replication (*ori*) [11], proceeds bidirectionally, and terminates in the terminus region (*ter*) located opposite to *ori* [12]. Following replication termination, daughter chromosomes are separated by the action of topoisomerases to resolve catenanes and site specific recombinases at the *dif* site in the *ter* region to resolve chromosome dimers [13], [14].

A large fraction of sequenced bacterial genomes encodes orthologs of the ParA and ParB proteins [15], which have been shown to be involved in chromosome segregation in several species [2]. Yet, several bacterial species – notably *Escherichia coli* - lack ParAB orthologs. Similarly, in *Bacillus subtilis* the ParA homolog Soj is not essential for chromosome segregation [16] and its absence causes only minor defects in chromosome segregation [17]. Similarly to plasmid-encoded *parAB* systems, the chromosomally encoded *parAB* systems consist of two proteins, ParA and ParB, and the centromere-like *parS* site [18], which is located in the *ori*-proximal region of the chromosome and consists of repeated *parS* sequences [15]. ParB proteins bind to the *parS* sequences [15], [19]–[23]. ParA proteins are P-loop ATPases that interact with the ParB/*parS* complex [24], [25]. *In vitro* ParA proteins form filaments in an ATP-dependent manner and their weak ATPase activity is stimulated by ParB [24], [25]. Moreover, ParA proteins have been reported to bind non-specifically to DNA [25]–[27]. The function of the *parABS* system in chromosome segregation is best understood in the alphaproteobacterium *Caulobacter crescentus* and for chromosome I in the gammaproteobacterium *Vibrio cholerae*. In these two bacteria, *ori* and the flanking *parS* site with bound ParB are located at an extreme cell pole while ParA is thought to form a filamentous structure over the chromosome. After duplication of *parS*, one ParB/*parS* complex remains at the original pole while the second complex is bound by ParA filaments and moved unidirectionally to the opposite pole in a manner that depends on ATP hydrolysis by ParA [24], [28], [29].

Much of the work on chromosome arrangement has focused on *C. crescentus* and the gammaproteobacteria *E. coli* and *Pseudomonas aeruginosa*. In *C. crescentus*, the chromosome is arranged about a longitudinal axis. As described, in newborn cells, *ori* is close to the old pole and the *ter* region close to the opposite pole [3]. After completion of replication and segregation, the predivisional cell contains two segregated chromosomes displaying mirror symmetry [3]. In *P. aeruginosa*, the chromosome is overall arranged as in *C. crescentus*; however, the *ori* and *ter* regions are localized at a distance from the extreme poles [9]. In slow-growing cells of *E. coli*, the single chromosome is arranged about a transverse axis with *ori* at midcell, *ter* broadly distributed around midcell, and the two replichores occupying separate cell halves in newborn cells [4]. During replication, the duplicated replichores segregate bidirectionally and symmetrically with the replicated *ori*'s segregating from midcell to ¼ and ¾ positions, ultimately generating a predivisional cell with the two segregated chromosomes displaying translational symmetry [4], [30]. In *E. coli* as well as in *C. crescentus* and *P. aeruginosa* daughter loci are sequentially and in a directed manner segregated to their final subcellular locations suggesting that the spatial arrangement of chromosomes is laid down during segregation [3], [4], [9].

Similarly to chromosome arrangement, the localization of the two sister replisomes varies between species: In *E. coli* the two replisomes, after assembling at *ori* in the midcell region, separate and move to opposite cell halves, return to midcell for termination, and then disassemble [10], in *B. subtilis*, a low GC Gram-positive bacterium, the replisomes assemble at *ori* near midcell but - although highly motile - the two replisomes are more restricted to the midcell area [31], [32]. In *P. aeruginosa* the replisomes also localize at midcell, however, in this species the chromosome is moved sequentially to midcell prior to replication [9]. Finally, in *C. crescentus* the replisomes, after assembling at the polarly localized origin, move jointly towards midcell [33].

Here, we focused on elucidating chromosome dynamics and spatial arrangement in the deltaproteobacterium *Myxococcus xanthus*. Cells of *M. xanthus*, in response to nutrient starvation, initiate a developmental program that culminates in the formation of spore-filled fruiting bodies [34]. Fruiting body formation as well as spore formation are closely coupled to cell cycle progression, i.e. DNA replication during the aggregation phase of fruiting body formation is essential to complete aggregation and sporulation [35], [36] and while rod-shaped vegetative cells contain one chromosome after cell division [37]–[39], mature spores contain two chromosomes [37]. To understand the coupling between cell cycle regulation and development, we focused on determining the spatial arrangement of the 9.1 Mb *M. xanthus* chromosome as well as chromosome dynamics during replication and segregation in vegetative cells. Our data provide evidence that *M. xanthus* uses a *parABS*-based mechanism for chromosome segregation and displays a novel spatial arrangement.

## Results

### Overall arrangement of the *M. xanthus* chromosome

The 9,139,763 bp *M. xanthus* genome is circular and with the predicted *ori* and *ter* located by GC nucleotide skew at position 1 and 4,547,166 (corresponding to 49.75% or 179° of the circular chromosome), respectively [40], [41] suggesting that DNA replication divides the circular *M. xanthus* chromosome into almost equal replichores. Close to *ter*, a putative *dif* site at position 4,489,697 bp (49.12% or 177° of the chromosome) was identified [41].

**Figure 1:**
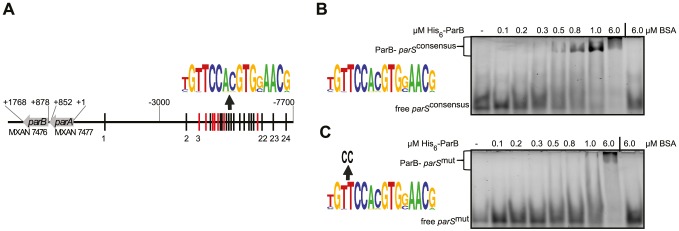
The *M. xanthus* genome encodes a *parABS* locus and contains 24 *parS* sequences in the *ori* proximal region. (A) The *parABS* locus. Seven *parS* sequences encoded on the - strand are indicated in red, the remaining 17 are indicated in black. A weblogo (version 2.8.2; http://weblogo.berkeley.edu/) based on the 24 *parS* sequences is shown above. Coordinates.of the *parABS* locus are relative to the start codon of *parA*. (B, C) Electrophoretic mobility shift assays of DNA binding by His_6_-ParB. A double-stranded 5′-HEX-labelled 26 bp oligonucleotide pair containing a consensus *parS* site (weblogo on the left, B) or a *parS* site with two substitutions (weblogo on the left, C) (each 100 nM) was incubated without additives (lane 1), with increasing amounts of His_6_-ParB (lanes 2–7) or 6 µM BSA (lane 8). Free and protein bound DNA are indicated.

Bioinformatic analysis identified a *parAB* locus (MXAN7477 and MXAN7476) located in the vicinity of *ori* in the *M. xanthus* genome [42] (Figure 1A). Moreover, we previously identified 24 *parS* sequences in the region upstream of *parAB* (Figure 1A) [39]. The *parABS* locus localizes approximately 30 kb away from *ori*. The ParA protein encoded by MXAN7477 is a member of the ParA/MinD superfamily of P-loop ATPases and groups with other ParA proteins involved in chromosome segregation (Figure S1, S2). *M. xanthus* ParA contains the basic amino acid residues important for non-specific DNA binding of ParA (Soj) from *B. subtilis* and *C. crescentus* [24], [26] (Figure S3). The *M. xanthus* ParB homolog contains the conserved regions involved in dimerization and DNA-binding [22], [43]–[45] (Figure S4, S5, S6). Moreover, in the N-terminal part, a region around a basic residue, which is thought to be involved in activating the ATPase activity of the partner ParA protein [25] (Figure S6, region 1), is conserved.

### ParB binds to a consensus *parS* sequence *in vitro*

To determine whether *M. xanthus* ParB binds to *parS* sequences, we purified an N-terminal His_6_-tagged ParB variant to homogeneity (Figure S7). Subsequently, binding to a 26 bp hexachlorofluorescein-(HEX) labelled DNA fragment containing a *parS* consensus sequence (HEX-*parS* ^consensus^) by His_6_-ParB was tested using an electrophoretic mobility shift assay (EMSA). As shown in Figure 1B, His_6_-ParB bound to HEX-*parS* ^consensus^. Moreover, His_6_-ParB bound to a HEX-labelled 26 bp fragment containing a mutant version of the consensus *parS* sequence (HEX-parS^mut^) but with an approximately 10-fold reduced affinity compared to HEX-*parS* ^consensus^ (Figure 1C). Thus, ParB binds specifically to the consensus *parS* sequence *in vitro*.

### Dynamic localization of ParB-YFP *in vivo*

**Figure 2:**
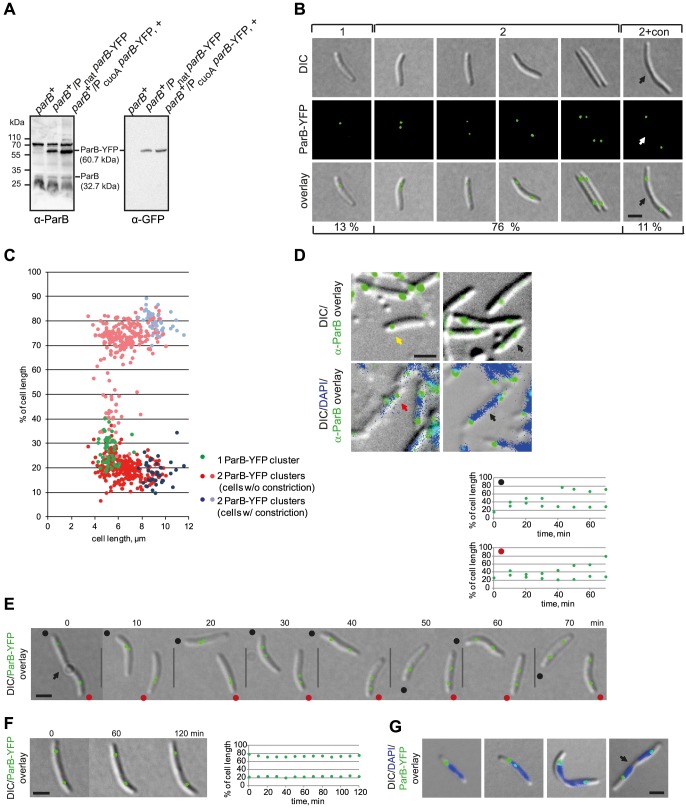
ParB-YFP localizes dynamically and in subpolar clusters. (A) Immunoblot analysis of ParB and ParB-YFP accumulation in cells of the indicated genotypes. Equal amounts of protein were loaded per lane and the blots were probed with α-ParB and α-GFP antibodies as indicated. ParB and ParB-YFP with their calculated molecular masses as well as positions of molecular markers are indicated. + indicates that the corresponding strain was grown in the presence of 150 µm CuSO_4_. (B) ParB-YFP forms either one or two clusters. Left panels show a representative short cell with one ParB-YFP cluster (1). The four panels in the middle show cells with two ParB-YFP clusters at different positions (2). The right panel shows a representative cell with a constriction and two ParB-YFP clusters (2+con). The constriction is indicated by an arrow. Scale bar, 2 µm. Numbers below indicate the percentages of cells with the indicated localization pattern (n = 335). (C) Quantification of ParB-YFP localization pattern. Diagram indicates cluster localization as % of cell length and as a function of cell length (n = 335). The old pole is at 0%. (D) Native ParB forms either one or two clusters. Immunofluorescence microscopy using affinity purified α-ParB antibodies (top and bottom rows) together with DAPI staining (lower row). The yellow arrow indicates a cell with a single cluster, black arrows indicate cells with two completely segregated clusters, the red arrow indicates a cell with two clusters, one of which is in an intermediate position. Scale bar, 2 µm. (E) Time-lapse images of ParB-YFP localization. The position of a constriction is indicated by the arrow. Because the recorded cells are moving, the old poles are indicated with a black and red dot. Diagrams above depict the positions of the ParB-YFP cluster in the two newborn cells (red and black dot) as % of cell length over time. The old pole is at 0%. Scale bar, 2 µm. (F) Time-lapse images of stationary ParB-YFP localization. The diagram on the right depicts the position of the ParB-YFP foci as % of cell length over time. Scale bar, 2 µm. (G) ParB-YFP localizes at the “edges” of nucleoids. The images show cells with one or two ParB-YFP cluster. The position of a constriction is indicated by an arrow. Scale bar, 2 µm.

ParB binding to *parS* sequences *in vivo* has been visualized in different bacteria using a fluorescent ParB fusion protein [21], [23], [24], [28], [46]–[49]. Therefore, to determine ParB localization in *M. xanthus in vivo*, we constructed the plasmid pAH7 encoding a ParB-YFP fusion. Because the ParB-YFP fusion did not complement a *parB* mutant (data not shown; see further below), pAH7 was integrated at the Mx8 phage *attB* site in the wild-type (WT) strain DK1622 giving rise to the merodiploid *parB* ^+^/*parB*-*YFP* strain SA4202. Immunoblot analysis using α-His_6_-ParB and α-GFP antibodies demonstrated that ParB-YFP accumulated at an elevated level compared to that of ParB (Figure 2A). Importantly, growth rate and cell size distribution of SA4202 were similar to WT (data not shown) demonstrating that the ParB-YFP fusion is not dominant negative.. In this strain, ParB-YFP formed well-defined clusters with 13% of cells containing a single cluster in a subpolar region and 76% containing two clusters, which either localized to the two subpolar regions or one cluster was in a subpolar region and the second in an intermediate position (Figure 2BC). The remaining 11% of cells contained a cell division constriction and had ParB-YFP localizing in clusters in the two subpolar regions. In cells with a single cluster, this cluster localized 1.4±0.3 µm from the nearest pole corresponding to 27%±6% of the cell length and in cells with two clusters in the subpolar regions, the clusters were at 20%±5% and 76%±4% of the cell length, respectively (Figure 2C). The pattern of ParB-YFP cluster localization correlated with cell length: Cells with a single cluster were short cells, cells with a subpolar and an intermediate cluster were slightly longer, and long cells including cells with a division constriction had ParB-YFP in the two subpolar regions (Figure 2BC). To verify that the ParB-YFP localization pattern reflects the localization of native ParB, despite its inability to complement a *parB* mutant, immunofluorescence microscopy using α-ParB antibodies was carried out on WT *M. xanthus* cells. As shown in Figure 2D, native ParB formed one or two clusters that generally localized to the subpolar regions of cells. In a few cells with two clusters, one was in a subpolar region and one in an intermediate position as observed with ParB-YFP. Because the ParB-YFP fusion localizes similarly to native ParB, we conclude that the ParB-YFP fusion is a valid tool to follow ParB localization. In total, the ParB localization data suggest that cells with a single subpolar ParB-YFP cluster are cells in which the *parS* sequences have either not replicated or not segregated, cells with a subpolar and an intermediate cluster represent cells in which one ParB-YFP bound to *parS* is segregating, and cells with two subpolar clusters represent cells in which the ParB/*parS* complex has completed segregation.

To test this idea, time-lapse fluorescence microscopy was performed. As shown in Figure 2E for representative cells, newborn cells contained a single ParB-YFP cluster in a subpolar region close to the old pole. 10 min after cell division, the ParB-YFP cluster splits into two clusters followed by asymmetric segregation of one of the clusters to the opposite subpolar region. Once segregation was complete, the two ParB-YFP clusters essentially remained fixed and only displayed minor movements (Figure 2F, representative cell). Averaging over several time-lapse recordings of segregating ParB/*parS* complexes, the ParB-YFP segregation process occurs over a period of 30–50 min and with an average speed of 0.05±0.03 µm/min, which is slightly lower that the speed observed in *B. subtilis* (0.17 µm/min) [5] and in *C. crescentus* (0.27 µm/min) [3].

To determine ParB-YFP localization with respect to the rest of the chromosome, we stained ParB-YFP expressing cells with DAPI (4′,6-diamidin-2-phenylindol) (Figure 2G). This analysis revealed that the single ParB-YFP cluster in short cells with one chromosome and the two ParB-YFP clusters in predivisional cells with two segregated chromosomes consistently localized at the extreme “edge” of a nucleoid. Only cells with a segregating ParB-YFP cluster had the cluster over a nucleoid (Figure 2G). Similarly, we observed by immunofluorescence using α-ParB antibodies that in WT *M. xanthus* cells stained with DAPI, native ParB localized at the extreme “edge” of a nucleoid in a subpolar region and that in a few cells one of the ParB clusters localized over a nucleoid (Figure 2D). We conclude that ParB/*parS* complexes with the nearby *ori* in *M. xanthus* localize in a subpolar region, and that large subpolar regions devoid of DNA separate ParB/*parS* complexes from the extreme poles.

### ParA-mCherry localizes mainly in dynamic subpolar patches

**Figure 3:**
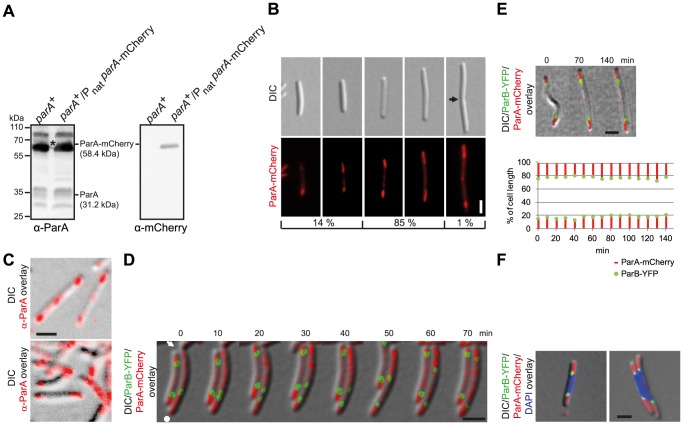
ParA-mCherry localizes dynamically and in subpolar patches. (A) Immunoblot analysis of ParA and ParA-mCherry accumulation in cells of the indicated genotypes. Equal amounts of protein were loaded per lane and the blots were probed with α-ParA and α-mCherry antibodies as indicated. ParA and ParA-mCherry with their calculated molecular masses are indicated. Positions of molecular markers are indicated. * Note that the band corresponding to ParA-mCherry is partially covered by a non-specific protein detected by α-ParA. (B) ParA-mCherry forms patches in the subpolar regions. Left panels, short cells with ParA-mcherry patches of unequal length and/or intensities. Next two panels, longer cells with ParA-mCherry patches of more equal length and intensities. Last panel, cell with a constriction, subpolar ParA-patches and ParA-mCherry at midcell. The position of a constriction is indicated by an arrow. Scale bar, 2 µm. The numbers below indicate the percentages of cells found with the indicated localization pattern (n = 100). (C) Native ParA forms patches in the subpolar regions. Immunofluorescence microscopy using affinity purified α-ParA antibodies. Scale bar, 2 µm. (D) Time-lapse images of ParB-YFP and ParA-mCherry localization. Cells were grown in the presence of 150 µM CuSO_4_. A constriction is indicated by the arrow. The old pole is indicated by the white dot. Scale bar, 2 µm. (E) Time-lapse movie of ParB-YFP and ParA-mCherry localization after completion of segregation. Cells were grown in the presence of 150 µM CuSO_4_. The diagram below depicts the position of the ParB-YFP clusters (green) and the subpolar ParA-mCherry patches (red) as % of cell length over time. Scale bar, 2 µm. (F) ParA-mCherry and ParB-YFP localize at the “edges” of a chromosome. Cells were grown in the presence of 150 µM copper. Scale bar, 2 µm.

ParA localization has been successfully analysed in different bacteria using C-terminal fluorescent fusion proteins [24], [28], [29], [50] Similarly to the ParB-YFP fusion, the ParA-mCherry fusion did not complement a *parA* mutant (data not shown; see further below). Therefore, the plasmid pAH59, which encodes the ParA-mCherry fusion, was integrated at the *attB* site of the WT giving rise to the merodiploid *parA* ^+^/*parA*-*mCherry* strain SA4255. Immunoblots using α-ParA and α-mCherry antibodies showed that ParA-mCherry accumulated at a slightly elevated level compared to that of ParA (Figure 3A). Importantly, growth rate and cell size distribution of SA4255 were similar to WT (data not shown) demonstrating that the ParA-mCherry fusion is not dominant negative.

ParA-mCherry displayed several different localization patterns that correlated with cell size. Notably, large patches of ParA-mCherry were often observed in the subpolar regions (Figure 3B). In 14% of cells, and these were especially shorter cells, the ParA-mCherry patches were of unequal length and/or intensities (Figure 3B) while in longer cells, corresponding to 85% of cells, the patches were of more equal length and intensities. Finally, in 1% of cells, and these were especially cells with constrictions, ParA-mCherry was observed at the incipient division site (Figure 3B). Generally, little ParA-mCherry was observed between the subpolar patches (Figure 3B, Figure 3D, left cell, Figure 3E). To verify that ParA-mCherry localization mirrors the localization of native ParA, immunofluorescence microscopy on WT *M. xanthus* cells using α-ParA antibodies was carried out. As shown in Figure 3C, native ParA formed patches in the subpolar regions of WT cells as described for ParA-mCherry and with relatively little ParA localizing between the subpolar patches. Because the ParA-mCherry fusion localizes similarly to native ParA, we conclude that the ParA-mCherry fusion is a valid tool to follow ParA localization.

To follow ParA-mCherry and ParB-YFP dynamics in parallel, we performed time-lapse microscopy on cells of SA5812 that expressed both fusion proteins. In this strain, ParA-mCherry was expressed as in SA4255 and ParB-YFP was expressed from the copper inducible *cuoA* promoter (P*_cuoA_*) at the *cuoA* locus. In the presence of 150 µM copper, ParB-YFP accumulated at a level similar to that in SA4202 (Figure 2A). We observed that in newborn cells, an intense ParA-mCherry patch extended from the old pole and up to the single ParB-YFP cluster, while a less intense ParA-mCherry cloud, similar to the ParA-YFP cloud in *C. crescentus* [29], extended from the new pole up to the single ParB-YFP focus (right cell in Figure 3D, representative cell). During the asymmetric segregation of one of the ParB-YFP complexes, the less intense ParA-mCherry cloud shortened but consistently extended from the pole up to the segregating ParB-YFP cluster. In parallel, the intensity of the retracting cloud at the new pole increased and ultimately appeared as a patch similar to the one at the old pole (70 min). In contrast, the ParA-mCherry patch at the old pole remained more or less constant in length and intensity (Figure 3D, right cell). During the segregation process, little ParA-mCherry was observed between the ParB-YFP clusters (Figure 3D, right cell, 10–60 min). Once the segregating ParB-YFP cluster had reached the subpolar region neither ParB-YFP nor ParA-mCherry localization changed significantly over time (left cell Figure 3D and Figure 3E, representative cells). As shown by DAPI staining, the high intensity subpolar ParA-mCherry patches extending from a pole up to ParB-YFP localized in the nucleoid free areas in the subpolar regions (Figure 3F).

Thus, ParB-YFP segregates in the wake of a shortening ParA-mCherry patch until the two proteins reach the “edge” of a chromosome in a subpolar region and from then on they remain essentially stationary.

### The *M. xanthus* chromosome is arranged around a longitudinal axis

**Figure 4:**
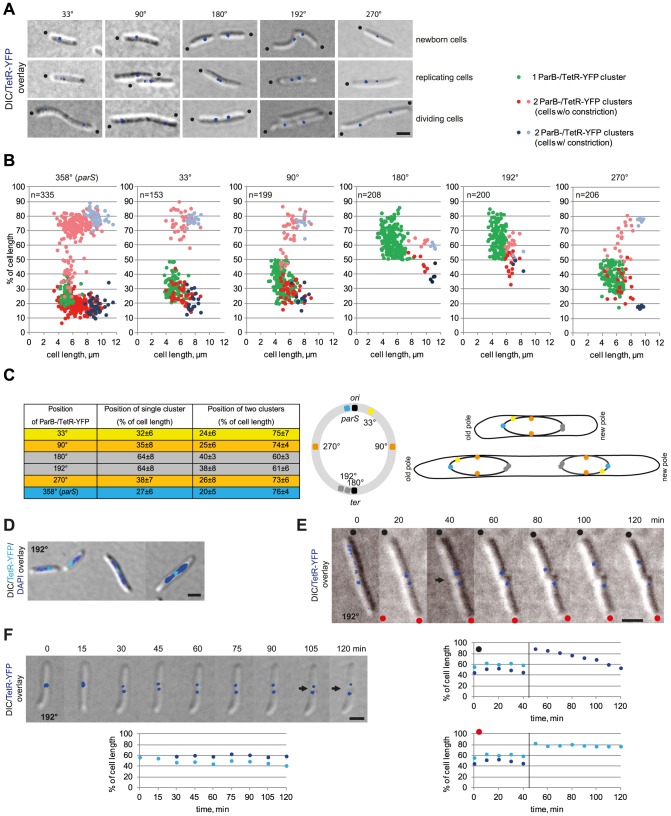
The *M. xanthus* chromosome is arranged about a longitudinal axis. (A) The localization of five chromosomal loci in *M. xanthus* cells. Images of newborn, replicating and dividing cells with one or two TetR-YFP clusters in strains with *tetO*-arrays inserted at the indicated positions on the chromosome were taken from time-lapse movies. Old poles are indicated by black dots. Scale bar, 2 µm. (B) Localization of five chromosomal loci in *M. xanthus* in comparison to ParB-YFP. Diagrams depict the positions of the TetR-YFP clusters in cells with a *tetO*-array inserted at the indicated positions on the chromosomes as % of cell length and a function of cell length (for each n∼200) in comparison to ParB-YFP localization (n = 335). The old pole represents 0% of cell length. (C) Summary of the average positions ± standard deviation (SD) for one or two TetR-YFP clusters in cells with a *tetO*-array inserted at the indicated positions on the chromosome. The cartoon shows the circular chromosome with the positions of the *tetO*-arrays using the colour code in the left panel. The right scheme depicts *ori*-*ter*, and *ori*-*ter*-*ter*-*ori* organisation in cells before (top) and after (bottom) chromosome replication and segregation using the colour code in the left panel. (D) TetR-YFP localization in cells with the *tetO*-array inserted at 192°. Scale bar, 2 µm. (E) Time-lapse images of the division of a cell with the *tetO*-array inserted at 192°. Old poles are labeled with black and red dots. The constriction is indicated by the arrow. Scale bar, 2 µm. Diagrams below show the positions of the TetR-YFP clusters in the two daughter cells over time. The old pole is at 0% of cell length. (F) Time-lapse images during replication of *ter* in cells with the *tetO*-array inserted at 192°. Scale bar, 2 µm. Diagram depicts the positions of the TetR-YFP clusters over time. The old pole is at 0% of cell length.

To determine the overall arrangement of the *M. xanthus* chromosome we used the fluorescent repressor operator system (FROS) in which an array of approximately 240 *tetO* operator sequences is inserted at specific locations on the chromosome and detected by the binding of a TetR-YFP protein [3], [4], [51], [52]. To adopt this system for *M. xanthus*, the *tetO*-array was cloned in five plasmids each containing one of five different *M. xanthus* chromosomal fragments. Subsequently, these five plasmids were integrated at the pre-defined chromosomal loci on the *M. xanthus* chromosome by single homologous recombination giving rise to a total of five strains containing the array at 33°, 90°, 180°, 192°, and 270° (Figure 4C). Subsequently, the plasmid pMat6, which allows expression of TetR-YFP from the *cuoA* promoter, was introduced into the Mx8 phage *attB* site of the five strains. In the presence of 150–300 µM copper, TetR-YFP did not form clusters in a strain that did not contain the *tetO*-array (data not shown), whereas distinct clusters were observed in the five *tetO*-array containing strains in the presence of 150–300 µM copper. TetR-YFP bound to the *tetO*-arrays may function as a replication roadblock and cause growth defects [2]. We only observed a slight growth defect for the strain SA4127 (*tetO*-array at 180°) in the presence of 150–300 µM copper (data not shown).

All five strains contained one or two TetR-YFP clusters in a reproducible pattern in the presence of 150–300 µM copper (Figure 4ABC). Quantitative analyses of snapshot images together with time-lapse recordings showed that single TetR-YFP clusters at the *tetO*-array at 33°, 90°, 180°, 192°, and 270° localized at 32±6%, 35±8%, 64±8%, 64±8% and 38±7% of the cell length, respectively. As expected, the frequency of cells with two clusters decreased the further away from *ori* the *tetO* array was inserted on the chromosome (Figure 4B). In cells with two clusters, the clusters generally localized symmetrically around midcell: The *tetO* array at 33° gave rise to clusters close to the subpolar regions, the *tetO* arrays at 182° and 190° to clusters close to midcell, and the *tetO* arrays at 90°, 270° to clusters at intermediate positions (Figure 4ABC). Moreover, time-lapse recordings showed that for all five loci, segregation was asymmetrical with one cluster remaining essentially stationary and the second copy reproducibly segregating in a directed manner to the opposite cell half (data not shown).

To determine the localization dynamics of the *ter* region (as indicated by TetR-YFP binding to the arrays at 180° and 192°), time-lapse microscopy on the strain containing the *tetO* array at 192° was performed. These analyses showed that immediately after a cell division, the *ter* region localized close to the subpolar region of the new cell pole and separated by 1.0±0.3 µm from the extreme pole (Figure 4E, representative cells). From this position, the *ter* region relocated towards the midcell region in a highly variable pattern, e.g. relocation of the *ter* region in the cell marked with a black dot in Figure 4E occurred in 60 min whereas localization of the *ter* region in the cell marked with a red dot was essentially unchanged in this time interval. Once in the midcell region, the *ter* region separated into two clusters that localized symmetrically around midcell (Figure 4F, representative cell).

DAPI staining of cells with the *tetO* array at 192° revealed that in short cells with a single TetR-YFP cluster, the *tetO*-array localized at the edge of the nucleoid in a subpolar region, whereas in longer predivisional cells it localized at midcell (Figure 4D). On the basis of these analyses, we conclude that the bulk of the *M. xanthus* chromosome in newborn cells is localized between the *ori* and *ter* regions and that these two regions are in opposite subpolar regions. Moreover, the *M. xanthus* chromosome is arranged around a longitudinal axis, with *ori* next to the subpolar region of the old pole and *ter* next to the subpolar region at the new pole. After replication, the two chromosomes are arranged about a transverse axis, showing mirror-symmetry with an *ori-ter-ter-ori* arrangement (Figure 4C). Also, our data suggest that after the segregating ParB/*parS* cluster has reached the opposite subpolar region, the remainder of the segregating daughter chromosome follows and is “laid down” between the segregated ParB/*parS* complex and the unreplicated mother chromosome. The irregular relocation of the *ter* region from a subpolar region to the midcell region suggests that the mechanism underlying this relocation is different from that driving the segregation of the replicated *ori*. We suggest that the segregation of one of the daughter chromosomes results in the *ter* region of the unreplicated chromosome gradually and passively being displaced to the midcell region. In total this segregation pattern results in two daughter chromosomes that are arranged about a transverse axis at midcell and showing mirror symmetry.

### The *M. xanthus* chromosome is replicated by independently moving replisomes

**Figure 5:**
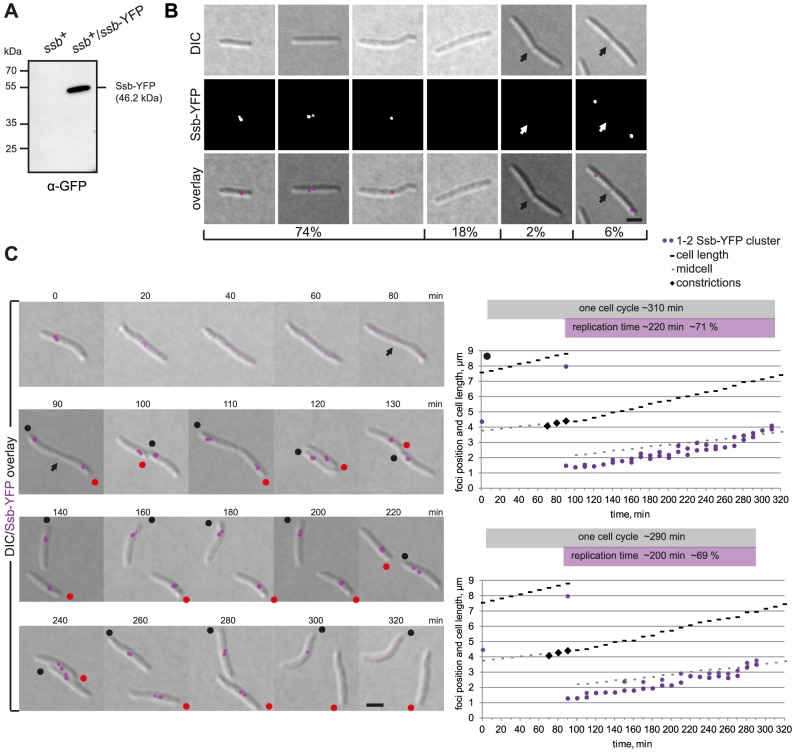
Ssb-YFP localization reveals two independently moving replisomes. (A) Immunoblot analysis of Ssb-YFP accumulation in cells of the indicated genotype. Equal amounts of protein were loaded per lane and the blots probed with α-GFP antibodies. Ssb-YFP with its calculated molecular mass as well as positions of molecular markers are indicated. (B) Ssb-YFP forms distinct clusters. The left three panels show cells with one or two Ssb-YFP clusters. The next two panels show long cells and cells with a constriction in which no signal can be detected. The right panel shows a representative cell with a constriction (arrow) with two Ssb-YFP foci close to the old poles in the incipient daughter cells. Scale bar, 2 µm. (C) Time-lapse images of a dividing cell and its two daughter cells completing a full replication cycle. Notice that the cells are moving and the old poles of the two daughter cells are labeled with black and red dots. Scale bar, 2 µm. Diagrams show the positions of the Ssb-YFP clusters in the two daughter cells (black and red spot) over time. Grey boxes indicate one cell cycle based on the time intervals between the disappearance of the Ssb-YFP signals (cell marked by black dot, 10–320 min; cell marked with red dot, 10–300 min). Purple boxes indicate the time intervals in which replication occurs based on the Ssb-YFP signals (cell marked with black dot, 90–310 min; cell marked with red dot, 90–290 min).

The previous analyses suggest that replication of the *M. xanthus* chromosome initiates early after cell division. To monitor the timing of replication, we generated a strain that allowed the localization of single-strand binding protein (Ssb; MXAN1071) and could be used as a proxy for replisome localization. Ssb binds to unwound DNA on the lagging strand during replication [53] and Ssb has previously been used to follow the localization and dynamics of the replisomes in *E. coli* [10]. The full-length *ssb* gene including its promoter was fused to YFP in the plasmid pEB8 and integrated by single homologous recombination in the *ssb* gene giving rise to the the *ssb* ^+^/*ssb*-YFP merodiploid strain SA5007. Immunoblot analyses with α-YFP antibodies confirmed that a Ssb-YFP protein of the expected size accumulated in this strain (Figure 5A). Growth rate and cell size distribution of SA5007 were similar to WT (data not shown).

Snapshot analysis showed that cells either contained a single Ssb-YFP cluster (45%), two clusters (29%), or no clusters (20%). Cells with no Ssb-YFP clusters were enriched in long cells and in cells with cell division constrictions (Figure 5B). Moreover, cells with deep constrictions contained two Ssb-YFP clusters in the subpolar regions close to the old poles (6%) (Figure 5B). To follow the dynamics of the replisomes, we monitored Ssb-YFP localization by time-lapse microscopy (Figure 5C, representative cells): In dividing cells, the reappearance of Ssb-YFP clusters was observed shortly before the two daughter cells physically separated (90 min). Subsequently, in both daughter cells, the Ssb-YFP cluster(s) moved from the old pole towards midcell alternating in an apparently random pattern between one and two clusters suggesting that the two replisomes merge and split. The replisomes disassembled in the midcell region 200–220 minutes after their appearance and 80–100 min before cell division. The splitting and merging of the Ssb-YFP clusters suggest that the two replisomes move independently of each other and that the merging events may be the result of the two replisomes moving close together and appearing as a single cluster before moving apart, resulting in the single cluster splitting into two. The speed of Ssb-YFP movement was 0.78±0.12 µm/h and slightly faster than the cell elongation rate of 0.72±0.06 µm/min (Figure 5C). However, the speed of Ssb-YFP movement occasionally exceeded the speed of cell elongation ∼1.4-fold, suggesting that movement of the replisomes is due to tracking on the chromosomes and not due to cell elongation. In *C. crescentus*, the speed of replisome movement as measured using a HolB-GFP fusion is 0.45±0.11 µm/h and exceeds cell elongation (0.34±0.08 µm/h) only ∼1.3 fold [33].

### ParB is essential for chromosome segregation

**Figure 6:**
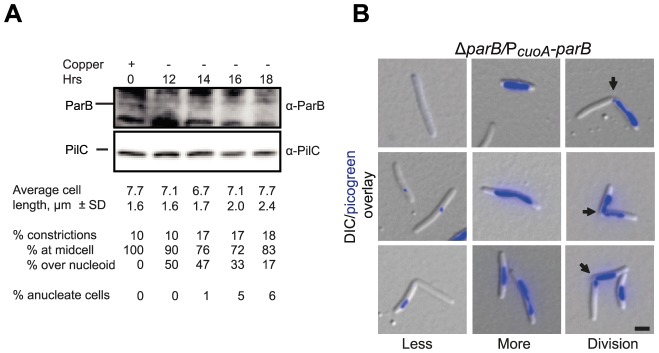
ParB is essential in *M. xanthus*. (A) Depletion of ParB. Upper panel, immunoblot analysis of the level of ParB during ParB depletion in SA4269 (Δ*parB*/P*_cuoA_*-*parB*). Cells were transferred to copper-free medium at t = 0 hrs. The two upper panels show the levels of ParB and PilC during ParB depletion. PilC is involved in type IV pili function [78] and is used as a loading control. Average cell length ± SD, % of cells with constrictions, and of those % of constrictions at midcell and/or over the nucleoid, and % of anucleate cells (n = 100 at each timepoint) are indicated. Cells had a doubling time of 5.5 h in the presence of CuSO_4_ and in the absence of CuSO_4_ until 16 hrs. (B) Depletion of ParB causes chromosome segregation and cell division defects. Left panel, anucleate cells or cells with only low levels of DNA stain (blue) (Less). Middle panel, cells with unusually bright DNA stains (More). Right panel, cells with constrictions (arrows) at the edge of chromosomes or over the chromosomes (Division). Images were taken at t = 18 h. Scale bar, 2 µm.

ParB in *C. crescentus* [54] and ParB for chromosome II in *V. cholerae* [55] are essential. To test whether ParB is essential in *M. xanthus*, we attempted to generate an in-frame deletion of *parB* using standard methods [56]. However, we were unable to generate this deletion unless a second copy of *parB* was present (data not shown). Similarly, we were unable to generate a *parA* in-frame deletion (data not shown). These observations show that ParB is essential and suggest that ParA is also essential for viability. To investigate the lethality of a *parB* null mutation, we cloned *parB* downstream from the copper inducible P*cuoA* promoter in a plasmid that was subsequently integrated at the chromosomal Mx8 phage *attB* site as recently described [57]. In this strain, an in-frame deletion of *parB* at the native site was generated giving rise to the strain SA4269 in which the single copy of *parB* present was expressed from P*cuoA*. Similarly to WT, SA4269 cells grew with a doubling time (5.5 h) in CTT medium supplemented with 300 µM copper. After removal of copper, ParB levels had decreased 4-fold by 14 h and were then undetectable by immunoblot analysis using α-ParB antibodies (Figure 6A). We conclude that the P*cuoA* construct allows the tight control of *parB* expression.

In agreement with the data, suggesting that *parB* is essential for viability, SA4269 formed colonies similar to WT in the presence of 150–300 µM copper and was unable to form colonies in the absence of copper. Importantly, following 16 h of ParB depletion in SA4269 and when ParB was not longer detectable by immunoblot analysis (Figure 6A), a decrease in growth rate was evident (data not shown). Microscopy of nucleoid-stained cells after 18 h of ParB depletion showed that the growth defect was accompanied by the formation of anucleate cells, cells containing only small amounts of DNA, cells with unusually brightly staining nucleoids and cells with a cell division constriction over a nucleoid; however, filamentous cells were not observed (Figure 6B). On the basis of these observations we concluded that lack of ParB causes severe chromosome segregation defects. The observation that ParB depletion does not result in the formation of filamentous cells but rather results in cells with division sites over a nucleoid and the formation of cells containing small amounts of DNA that presumably arise by division over a nucleoid and guillotining of the chromosome demonstrate that ParB is also essential for correct placement of the division site but not for cell division *per se*.

## Discussion

**Figure 7:**
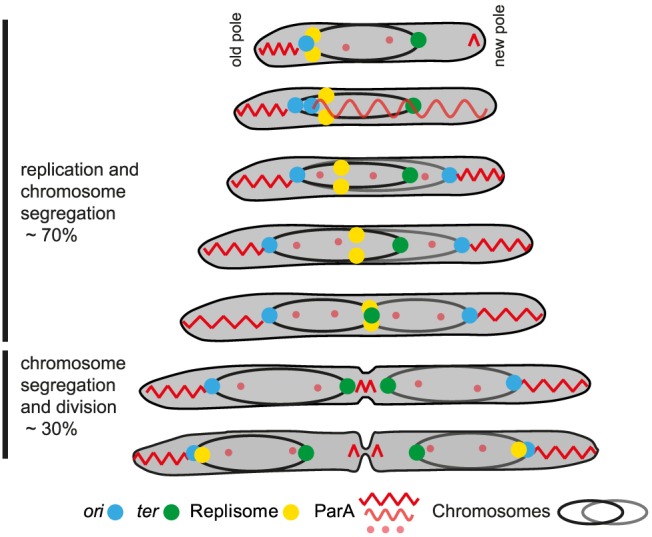
Chromosome arrangement and dynamics in *M. xanthus*. The diagram illustrates a cell through seven stages of chromosome replication, chromosome segregation and division during a cell cycle. The two chromosomes with their associated *ori* (blue dot) and *ter* (green dot) are shown in black and grey, respectively. The replisomes are shown as yellow dots. The different forms of ParA are shown in light and dark red. See text for a detailed description.

Here, we analysed chromosome arrangement and dynamics in the rod-shaped vegetative *M. xanthus* cells. Based on the determination of the subcellular location of six genetic loci and their segregation patterns, we conclude that the 9.1 Mb *M. xanthus* chromosome is arranged about a longitudinal axis (Figure 7). In newborn cells with a single chromosome, *ori* is in the subpolar region of the old pole and *ter* in the subpolar region of the new pole. The bulk of the chromosome is localized between *ori* and *ter*. Moreover, our data suggest that loci on both replichores are positioned at specific cellular locations and with these locations correlating with their location along the chromosome. As expected, replication and segregation occur concomitantly in *M. xanthus*. Our data suggest that during chromosome segregation, one sister chromosome remains essentially stationary whereas the second sister chromosome is segregated asymmetrically and unidirectionally to the opposite cell half. Specifically, the segregated *ori* region (as determined following the ParB/*parS* complex) is positioned in the subpolar region in the opposite cell half. Subsequently, genetic loci on the segregating daughter chromosome are laid down progressively and sequentially at their subcellular locations between this subpolar region and midcell. In parallel, and likely as a consequence of the segregating sister chromosome being refolded into compact chromosomal domains thereby excluding the mother chromosome, as has been proposed for *C. crescentus* [33], the *ter* region of the mother chromosome is displaced towards midcell. Thus, after replication and segregation are complete the two chromosomes in the predivisional cell display mirror symmetry about an axis at midcell and with the two *ori*'s in the two subpolar regions and the two *ter* regions symmetrically localized around midcell (Figure 7).

*M. xanthus* chromosome arrangement and dynamics combines features known from other bacteria with novel, unique features. Similar to the *C. crescentus* and *P. aeruginosa* genomes [3], [9], the *M. xanthus* chromosome is arranged about a longitudinal axis in newborn cells and predivisional cells contain two chromosomes displaying mirror symmetry. However, in *C. crescentus*, the ParB/*parS* complex is at the extreme pole and anchored to this pole by the PopZ protein [58], [59] and the *ter* region is close to the opposite pole [3] whereas the ParB/*parS* complex and *ter* region in *M. xanthus* and *P. aeruginosa* [9] are localized in the subpolar regions and in *M. xanthus* with a distance to the nearest poles of approximately ∼1 µm in newborn cells. In sporulating *B. subtilis* cells, the *ori* regions are also at the extreme poles and anchored to the poles by RacA, which is, in turn, anchored by DivIVA [60], [61]. Similarly, in the actinobacterium *Corynebacterium glutamicum* the ParB/*parS* complex is anchored to the cell poles by DivIVA [62]. The *M. xanthus* genome neither encodes a PopZ nor a RacA homolog. *M. xanthus* contains a DivIVA homolog, however, an in-frame deletion of this gene neither affects cell division [39] nor chromosome segregation or arrangement (data not shown). The reproducible localization of the ParB/*parS* complex in *M. xanthus* suggests that a factor analogous to that of PopZ, RacA and DivIVA functions to anchor the ParB/*parS* complex in the subpolar regions. As opposed to PopZ, RacA and DivIVA, we predict that this factor is not only polarly localized but extends from the pole essentially functioning as an outpost of the pole.

ParA localizes in a highly dynamic pattern in *M. xanthus*. In newborn cells, ParA-mCherry formed a high intensity patch in the subpolar region of the old pole that extended from the extreme pole up to the ParB/*parS* complex (Figure 7). In contrast the new pole had only a short patch of ParA at the pole. During ParB/*parS* segregation, ParA formed a cloud that extended all the way from the new pole over the nucleoid to the segregating ParB/*parS* complex. During the segregation process, this cloud shortened and the segregating ParB/*parS* complex followed in the wake of the shrinking ParA cloud. After completion of ParB/*parS* segregation, ParA-mCherry localized to high intensity patches in the two DNA free subpolar regions extending from the poles up to the ParB/*parS* complexes (Figure 7). Finally, late during cell division, ParA also accumulated in the midcell area.

Based on analyses of the *parABS* system in *C. crescentus* and *V. cholerae* for chromosome I, ParA has been suggested to form a filamentous structure over the chromosome [24], [28], [29]. Interaction of filamentous ParA with ParB in the ParB/*parS* complex is thought to cause activation of ParA ATPase activity resulting in the depolymerization of the filamentous ParA structure and the movement of the ParB/*parS* complex at the trailing end of the depolymerizing ParA structure. The data reported here for the dynamic movements of ParB and ParA are consistent with this model. Following completion of ParB/*parS* complex segregation in *C. crescentus* and *V. cholerae*, ParA relocates from the pole to form a cloud over the chromosomes [24], [28], [29]. Interestingly, we did not observed this relocation for ParA in *M. xanthus*. Rather ParA mostly remained in the nucleoid free subpolar regions after completion of ParB/*parS* segregation and it was not until late in the cell cycle that ParA accumulated at the incipient division site, in this way preparing for the next round of ParB/*parS* segregation. The ParA cloud in *M. xanthus* is only formed in newborn cells after initiation of replication. It will be interesting to explore how formation of this ParA cloud occurs in *M. xanthus* and how it is regulated.

In *V. cholerae*, HubP interacts with ParA and anchors it to the poles [63]. In *C. crescentus*, TipN interacts with ParA at the new pole and helps to maintain directionality in the ParB/*parS* segregation process [24], [29]. *M. xanthus* neither encodes a HubP nor a TipN homolog. We suggest that *M. xanthus* contains an analog of HubP and TipN and that this protein functions to organize/tether ParA in the subpolar regions. As reported for TipN [24], this analog could also be responsible for the relocation of ParA to the future new pole late in the cell cycle. It is tempting to speculate that this protein could be identical to the protein that helps to anchor ParB to a subpolar region. Alternatively, two different proteins function in unison to anchor ParB and ParA. ParA proteins form filaments *in vitro* in an ATP-dependent manner [64]–[67], bind non-specifically to DNA [25]–[27], [66] and are thought to form DNA associated filaments [24], [28], [29], [68]. An interesting question to address will be whether *M. xanthus* ParA also forms filaments in the nucleoid free subpolar regions. It is not clear why it would be advantageous for *M. xanthus* to keep the large subpolar regions chromosome free and to localize *ori* and *ter* to subpolar positions. Of note, the cell poles in *M. xanthus* serve as hubs for several dynamically localized motility proteins [69]. Keeping the poles free of DNA could potentially allow the unhindered dynamics of these proteins.

By following the localization of an Ssb-YFP fusion and using this fusion as a proxy for the replisomes, we observed that the replisomes assemble at *ori* in the subpolar region close to the old pole immediately prior to cell division. The two replisomes move slowly towards *ter*, which is in parallel displaced towards midcell from the opposite pole (Figure 7). Replication of *ter* occurs at midcell and the replisomes disassemble well ahead of cell division. In time-lapse recordings, Ssb-YFP in individual cells was observed to alternate between forming a single cluster and two well separated clusters. We speculate that this reflects that the two replisomes move on the DNA independently of each other and that a merging event represents two Ssb clusters merging to form a single cluster because the replisomes are in close physical proximity, while tracking independently on the two replichores. Similarly, splitting events would reflect that the two replisomes are moving apart from each other while tracking independently on each replichore.

The *M. xanthus* replisome dynamics share a unique blend of characteristics known from *C. crescentus* and *E. coli*. Similar to the replisomes in *E. coli* [10], the two replisomes in *M. xanthus* track independently along the two replichores and do not form a single “moving factory” as reported for *C. crescentus* [33]. However, the overall movement of the two replisomes in *M. xanthus* from a subpolar region towards midcell is similar to that in *C. crescentus* and unlike that in *E. coli* [10] in which the replisomes track along replichores localized in the two cell halves.

Our data suggest that replication in *M. xanthus* occurs during ∼70% of the cell cycle and starts immediately before cell division. Also, cells only undergo one round of replication per cell cycle. These observations are in agreement with previous analyses showing that chromosome replication in *M. xanthus* occurs through approximately 80% of the cell cycle as determined by quantitative autoradiography of pulse-labelled cultures [38]. Using this method a DNA synthesis rate of 25 kb/min was calculated [38]. Our data suggest a slightly lower replication rate of ∼22 kb/min, which is similar to the rate of 21 kb/min as determined for *C. crescentus*, which also undergoes only one round of replication per cell cycle [70].

Lack of ParB or of the cognate *parS* sequences has been shown to cause chromosome segregation defects including the formation of anucleate cells in *B. subtilis*, *C. glutamicum*, *P. aeruginosa, Streptococcus pneumonia, C. crescentus and V. cholerae* [9], [16], [21], [22], [28], [54], [55], [71]. However, ParB has only been demonstrated to be essential in *C. crescentus* and in *V. cholerae*. Specifically, lack of ParB in *C. crescentus*, in addition to chromosome segregation defects, causes the formation of filamentous cells and cell division defects [23]. The latter are likely caused by mislocalization of MipZ, which normally binds to ParB and inhibits FtsZ polymerization and cell division at the cell poles [72]. Similarly, ParBII acting on chromosome II in *V. cholerae* is essential and lack of ParBII, in addition to causing chromosome segregation defects, has been speculated to cause cell death by a mechanism that involves activation of toxin-antitoxin systems encoded on chromosome II [55]. ParB in *M. xanthus* is also essential and lack of ParB gives rise to chromosome segregation defects. However, unlike *C. crescentus* and *V. cholerae* lacking ParB and ParBII, respectively, *M. xanthus* cells that lack ParB frequently divide over a nucleoid thereby guillotining chromosomes. It thus seems that the molecular mechanisms underlying the essential nature of ParB are different in *C. crescentus*, *V. cholerae* and *M. xanthus*. We recently showed that the PomZ protein in *M. xanthus* is a positive spatial regulator of cell division and is essential for recruiting FtsZ to the incipient cell division site while PomZ does not affect chromosome segregation [39]. Moreover, we have proposed that localization of PomZ to midcell before FtsZ is guided by completion of either chromosome replication or segregation. Therefore, we suggest that the cell division defect in the absence of ParB in *M. xanthus* is caused by mislocalization of PomZ. In the absence of ParB, PomZ would not be directed to midcell but remain over a chromosome and, therefore, would recruit FtsZ and the remaining components of the divisome to a site that overlaps with a chromosome in this way giving rise to cell divisions over the nucleoid.

## Materials and Methods

### Bacterial strains, cell growth and strain construction

*M. xanthus* strains and plasmids are listed in Table 1 and 2, respectively. *E. coli* strains were grown in LB broth or 2×YT medium for protein overexpression [73]. Plasmids were propagated in *E. coli* TOP10 (F-, *mcrA*, Δ(*mrr-hsd*RMS-*mcr*BC), φ80*lac*ZΔM15, Δ*lac*X74, *deo*R, *rec*A1, *ara*D139, Δ(*ara, leu*) 7679, *gal*U, *gal*K, *rps*L, *end*A1, *nup*G) unless otherwise stated. *M. xanthus* strains were grown at 32°C in CTT media or on CTT agar plates with kanamycin and oxytetracycline (40 and 10 µg/ml, respectively) [74] unless otherwise stated. To regulate expression from the *cuoA* promoter, CuSO_4_ was added in concentrations between 150 µM and 300 µM.

**Table 1:** *Myxococcus xanthus* strains used in this work.

Strain	Relevant characteristics^a^	Reference
DK1622	Wild type	[81]
SA4202	*parB^+^*/P*_nat_*-*parB*-YFP (pAH7)	[39]
SA4255	*parA^+^*/P*_nat_*-*parA*-mCherry (pAH59)	This study
SA5812	*parAB^+^*/P*_nat_*-*parA*-mCherry (pAH59)P*_cuoA_-parB*-YFP (pAH73)	This study
SA4212	*tetO*-array in mxan0733 region (pNV3)	This study
SA4129	*tetO*-array in mxan1968 region (pMAT20)	This study
SA4125	*tetO*-array in mxan3779 region (pMAT19)	This study
SA4115	*tetO*-array in mxan4000 region (pMAT13)	This study
SA4118	*tetO*-array in mxan5499 region (pMAT18)	This study
SA4227	*tetO*-array in mxan0733 region (pNV3), P*_cuoA_*-*tetR*-YFP (pMAT6)	This study
SA4136	*tetO*-array in mxan1968 region (pMAT20), P*_cuoA_*-*tetR*-YFP (pMAT6)	This study
SA4127	*tetO*-array in mxan3779 region (pMAT19), P*_cuoA_*-*tetR*-YFP (pMAT6)	This study
SA4114	*tetO*-array in mxan4000 region (pMAT13), P*_cuoA_*-*tetR*-YFP (pMAT6)	This study
SA4122	*tetO*-array in mxan5499 region (pMAT18), P*_cuoA_*-*tetR*-YFP (pMAT6)	This study
SA5007	*ssb^+^/ssb*-YFP (pEB8)	This study
SA4269	Δ*parB*/P*_cuoA_*-*parB* (pAH57)^b^	This study

a Plasmids mentioned in parentheses contain the indicated alleles and were integrated at the Mx8 *attB* site, at the *cuoA* locus or other genomic loci when indicated. In P*_nat_* and P*_cuoA_* constructs, the corresponding genes were transcribed from the relevant native promoter or the copper-inducible P*cuoA* promoter, respectively.

b The in-frame deletion of *parB* extends from 19 to 851 relative to the start codon of *parB*.

**Table 2:** Plasmids used in this work.

Plasmid	Relevant characteristics^a^	Reference
pKA52	Overproduction of His_6_-ParB (based on pET45b(+))	This study
pAH7	P*_nat_*-*parB*-YFP, Mx8 *attB*, (P*_nat_* 1097 bp)^b^	[39]
pAH59	P*_nat_*-*parA*-mCherry, Mx8 *attB*, (P*_nat_* 1097 bp)^b^	This study
pAH73	P*_cuoA_-parB-YFP*, copper-dependent expression of *parB-YFP* c	This study
pNV3	*tetO*-array+fragment of mxan0733 region (33°)	This study
pMAT20	*tetO*-array+fragment of mxan1968 region (90°)	This study
pMAT19	*tetO*-array+fragment of mxan3779 region (180°)	This study
pMAT13	*tetO*-array+fragment of mxan4000 region (192°)	This study
pMAT18	*tetO*-array+fragment of mxan5499 region (270°)	This study
pMAT6	P*_cuoA_*-*tetR*-YFP, Mx8 *attB*	This study
pEB8	*P_nat_-ssb*-YFP for integration at native site (*P_nat_* 303 bp)^d^	This study
pAH57	P_cuoA_-*parB*, copper-dependent expression of *parB*, Mx8 *attB*	This study
pAH18	Construct for in-frame deletion of *parB*	This study
pAH17	Overproduction of His_6_-ParA (based on pET45b(+))	This study
pMAT15	Vector for copper-dependent expression^c^	This study
pLAU53	*lacI*-ECFP and *tetR*-YFP	[51]
pLAU44	*tetO*-array (240 copies of 19 bp *tetO* operator)^e^	[51]

a Promoters for the expression of a particular construct are indicated together with the integration site on the chromosome.

b The promoter P_nat_ comprises 1097 bp upstream of the *parA*-gene.

c The plasmid integrates at *cuoA* locus.

d The promoter P_nat_ comprises 303 bp upstream of the *ssb*-gene.

e The operators are separated by a 10 bp random sequence.

The in-frame deletion of *parB* in SA4269 (deletion extends from 19 to 851 relative to the start codon *parB*) was generated using standard methods [56]. Plasmids pAH57, which contains *parB* downstream of the *cuoA* promoter, was integrated at the *attB* site. To obtain the in-frame deletion of *parB*, cells were grown in CTT medium supplemented with tetracycline to select for maintenance of pAH57 and 300 µM copper sulfate to induce expression of *parB* from the *cuoA* promoter.

Plasmids containing the Mx8 *attB* site were integrated by site-specific recombination at the *attB* site other plasmids were integrated by homologous recombination. Strains containing plasmids integrated at the *attB* site, the *cuoA* and other loci were constructed by electroporation of plasmids into the relevant strains. All strains were verified by PCR.

#### Plasmid construction

All DNA fragments generated by PCR were verified by sequencing. Primers used are listed in Table S1. To generate the ParB overexpression plasmid pKA52, the *parB* gene was amplified using the primers DB01/DB02 and DK1622 genomic DNA as template giving rise to *parB* full length. The PCR product was then digested using BamHI and AvaI and cloned into pET45b^+^ (Novagen). To create pAH59, first the plasmid pAH3, containing the *parA* promoter and gene was constructed, by ligation of a PCR fragment amplified with KA436/KA437 into the vector pSWU30 [75] using EcoRI and BamHI. The plasmid pAH3 was treated with BamHI and HindIII to clone an equally treated mCherry gene fragment (mCherry fwd/mCherry rev) amplified on pMT935 [76] and to construct pAH59. The plasmid pAH59 allows expression of a ParA-mCherry fusion protein, in which the two proteins are linked by a 5 amino acid linker (GSAGS). To create pAH73, a new vector pMAT15, which allows cloning of genes with their ATG startcodon into a NdeI-site downstream of the P*cuoA*-promoter, was constructed first. Using chromosomal DNA from *M. xanthus* and the primers cuoApromoter-NdeI/cuoA+2 a 980 bp long *cuoA*-promoter fragment was amplified and blunt-ligated into the vector pCR-Blunt II -TOPO (Invitrogen). The obtained plasmid, with the *cuoA*-promoter oriented the same way as the two genes conferring resistance to Kanamycin and Zeocin was called pMAT15. To generate plasmid pAH73, a *parB*-*yfp* fragment was amplified with primers AH81/AH82 from plasmid pAH07. The product was cut with NdeI and XbaI and ligated into equally digested pMAT15.

The five different chromosomal fragments for the FROS system were generated with the following primer pairs on chromosomal DNA: mxan5499NheIR/mxan5499HindIIIF, mxan4000NheIR/mxan4000HindIIIF, mxan3799NheIR/mxan3799HindIIIF, mxan1968NheIR/mxan1968HindIIIF, mxan0733NheIR/mxan0733HindIIIF. The products were digested with NheI and HindIII. In parallel the plasmid pLAU44 [51] was digested with XbaI and NheI to generate the *tetO* array-Gmr–*tetO* array fragment. The *tetO* array fragment was ligated together with each of the five myxobacterial fragments into pBJ114 [77], which was digested with XbaI and HindIII, to generate the plasmids pNV3, pMAT20, pMAT19, pMAT13 and pMat18.

To generate pMAT6, a *tetR*-*yfp* fragment was amplified with primers tetR-YFP-F/tetR-YFP-R on plasmid pLAU53 [51]. The product was cut with NdeI and XbaI and ligated into equally digested pMAT3 [57]. The plasmid pEB8 consists of three independently amplified fragments. The fragment downstream of the *ssb*-gene was amplified with primers EB7/EB8 and chromosomal DNA. The product was digested with EcoRI and BamHI. The *yfp*-gene was amplified with primers EB11/EB12: The product was digested with XbaI and BamHI. The fragment containing the *ssb* gene, a part of its upstream region (303 bp), as well as a linker (encoding 10 aa: GWLRCFWRI) was amplified with the primers EB5/EB6.The product was digested with HindIII and XbaI. In pEB08 the three fragments are ligated between the HindIII and BamHI site of pBJ114 [77] in the order *ssb* upstream region –*ssb* gene- 10 aa linker-*yfp*-gene-*ssb* downstream region.

To generate the plasmid pAH57 the primers AH46/AH47 were used for amplification of full length *parB*. The PCR product was then digested with XbaI and BamHI and cloned into pMAT3 [57] which contains the *cuoA*-promoter. To generate the in-frame deletion of *parB*, the plasmid pAH18 was generated, by cloning an upstream region (AH13/AH14) and downstream region of *parB* (AH15/AH16) into the vector pBJ114 [77]. To generate the ParA overexpression plasmid pAH17, the full length *parA* gene was amplified using the primers KA456/KA457.The PCR product was then digested using BamHI and PstI and cloned into pET45b^+^ (Novagen).

### ParB depletion experiments

Strain SA4269 was grown at 32°C in CTT containing 300 µM CuSO_4_ and appropriate antibiotics. At an optical density at 550 nm of ∼0.5 cells were harvested (4000 rpm, 15 min, RT), washed with copper-free CTT medium and resuspended in copper-free CTT medium. Samples for western blot and microscopy analysis were taken immediately before and during the depletion experiment. The culture was grown with continuous dilutions in pre-warmed copper-free CTT medium to maintain exponential growth.

### Protein purification

To purify His_6_-ParB, Rosetta 2 DE3[F^−^ *ompT hsdS_B_* (r_B_^−^ m_B_^−^) *gal dcm*(DE3) (Novagen) containing pKA52 and pRARE2 (contains the tRNA genes *argU*, *argW*, *ileX*, *glyT*, *leuW*, *proL*, *metT*, *thrT*, and *argX*; Novagen) was grown in 2×YT supplemented 100 µg/ml ampicillin and 34 µg/ml chloramphenicol to mid-exponential phase at 37°C. Overexpression was induced with 0.5 mM IPTG and shift to 18°C over night. Cells were washed and resuspended in lysis buffer II (50 mM NaH_2_PO_4_, pH 8.0, 300 mM NaCl, 10 mM imidazole, 5 mM MgCl_2_, 0.1 mM EDTA, 100 µg/m PMSF, 10 U/mL DNaseI, 1 mM β-mercaptoethanol). After three passages through a French press (16000 psi), the suspension was centrifuged at 4°C for 30 min at 20000× g. Soluble His_6_-ParB was purified on a 1 ml Bio-scale mini profanity IMAC cartridge (BioRad) charged with Ni^2+^ ions and a 10 ml Bio-scale Mini Bio-Gel P-6 desalting cartridge (Profinia Native IMAC Purification Kit, BioRad). Fractions containing His_6_-ParB were dialyzed against 25 mM Hepes/NaOH, pH 7.2, 25 mM NaCl, 5 mM MgCl_2,_ 0.1 mM EDTA, 10% glycerol, 1 mM β-mercaptoethanol [72].His_6_-ParA was purified for antibody production using the same protocol but Rosetta 2 DE3[F^−^ *ompT hsdS_B_* (r_B_^−^ m_B_^−^) *gal dcm*(DE3) (Novagen) containing pKA17 and pRARE2.

### Immunoblot analysis

Rabbit antiserum against His_6_-ParA and His_6_-ParB were generated using standard procedures [73]. PilC antibodies were described previously [78]. Immunoblotting was performed using standard procedures [73] with rabbit α-ParA, α-ParB, and α-mCherry antibodies, mouse α-GFP antibodies and peroxidase-conjugated goat α-rabbit or α-mouse immunoglobulin G secondary antibodies as recommended by the manufacturer (Roche). Blots were developed using Luminata Western HRP substrate (Millipore). All gels for SDS-PAGE were loaded with samples containing equal amounts of total protein.

### Protein-DNA interaction analysis by EMSA

The two pairs of 26 nt complementary oligonucleotides of which one was HEX-labelled were annealed in annealing buffer (10 mM Tris-HCl, pH 8.0, 50 mM NaCl, 1 mM EDTA) by incubating the mixture (10 µM of each oligonucleotide) for 1 min at 94°C and a slow cool down to room temperature [72]. 5′-GAGGCTTGTTCCACGTGGAACGTCGG-3′ and 5′-CCGACGTTCCACGTGGAACAAGCCTC-3′ were used to form the DNA duplex *parS* ^consensus^ and 5′-GAGGCTTGCCCCACGTGGAACGTCGG-3′ and 5′-CCGACGTTCCACGTGGGGCAAGCCTC-3′ were used to form the DNA duplex *parS* ^mut^ (the underlined parts correspond to the sequences shown in Figure 1C and 1D). For every protein/DNA reaction (100 µl), 100 nM of the annealed pairs and 5 µM of unspecific competitor DNA (pBluescript II SK-) were incubated for 20 min at 30°C without or with the indicated amounts of His_6_-ParB or BSA (0.1–6 µM) using the binding buffer 38 mM Hepes/NaOH, pH 7,2, 38 mM NaCl, 5 mM MgCl_2_, 7% glycerol, 1 mm ß-mercaptoethanol [72]. Samples were separated for 5 h on a 6% PAGE at 4°C and 10 V/cm in 1×TBE buffer containing 5 mM MgCl_2_ [72]. For signal detection, a Typhoon 8600 imager (GE Healthcare) was used.

### Microscopy and data analysis

Cells from exponentially growing cultures were transferred to a slide with a 1.0% agarose pad buffered with TPM (10 mM Tris-HCl pH 7.6, 1 mM KHPO_4_ pH 7.6, 8 mM MgSO_4_) and covered with a coverslip [39]. For DAPI or Picogreen (Quant-iT PicoGreen, Molecular Probes) staining, cells were incubated with DAPI (1 µg/ml) or Picogreen (1/100 diluted) for 10 min prior to microscopy at 32°C. Cells were observed using a Zeiss AxioImager M1 fluorescence microscope equipped with a Cascade 1K CCD camera (Photometrics). Images were processed using Metamorph (Molecular Devices). For time-lapse recordings a temperature-controlled Leica DMI6000B microscope with an adaptive focus control equipped with a Prior Pro Scan motorized stage and a Hamamatsu Flash 4.0 camera was used. For time-lapse recordings cells were either applied to an agarose pad buffered with 0.1% CTT or cells were applied directly on a coverslip, which was then covered with a thick 0.1% CTT agarose pad to prevent dehydration during microscopy at 32°C. Images were processed using the Leica MM AF software package. Cell length (mean ± standard deviation) and fluorescence signals were quantified using Metamorph.

### Immunofluorescence microscopy

Immunofluorescence microscopy was performed as described [79] with the exception that cells were fixed using 1.3% paraformaldehyde and 0.008% glutaraldehyde and Alexa Fluor®594 conjugated goat anti-rabbit antibodies were used as secondary antibody. The α-ParA and α-ParB antibodies were affinity-purified using His_6_-ParA and His_6_-ParB as described by Lillie et al. [80].

## Supporting Information

Figure S1 Phylogenetic tree with the ParA protein (MXAN7477) from *M. xanthus* and selected ParA proteins. The tree shows two distinct groups: One comprising ParA proteins derived from plasmids and non-primary chromosomes (light grey shaded) and the other comprising ParA proteins derived from primary chromosomes (darkgrey shaded). A multiple sequence alignment of a manually selected set of ParA sequences was built using the l-ins-i algorithm of the MAFFT version 6.864b software package [82]. A ParA phylogenetic tree was constructed from the core region of that multiple sequence alignment corresponding to residues 23–272 of the *M. xanthus* ParA using FastTree version 2.1.4 with default settings [83]. Numbers on the tree indicate Shimodeira-Hasegawa local support values. The bar indicates the # of amino acids substitutions per site. The selected sequences are the following (accession no. from top to bottom): P07673 (IncC pRK2), YP_001711992 (SopA pVM01), NP_233494 (ParAII Vcho), NP_285325 (ParAII Deira), NP_051544 (ParAIII Deira), NP_232399 (ParAI Vcho), NP_742172 (ParA Pput), NP_422547 (ParA Ccre), YP_001289880 (ParA Mytu), NP_602287 (ParA Cglu), NP_628072 (ParA Scoe), NP_391977 (ParA Bsub), YP_635580 (ParA Mxan), NP_293739 (ParAI Deira).
(EPS)

Figure S2 Sequence identities of the ParA protein (MXAN7477) from *M. xanthus* and selected ParA proteins.The identity score matrix was generated with the BioEdit Sequence Alignment Editor software (version 7.1.3.0) based on the full-length alignment of selected sequences as described in Figure S1 and with non-identical sequences score zero and identical sequences score 1. Fields are shaded based on the identity score. Score = 1 dark-grey, score >0.5 grey, score >0.4 light-grey.
(EPS)

Figure S3 Alignment of the ParA protein (MXAN7477) from *M. xanthus* and selected ParA proteins. Sequences are the same as in Figure S1. Residues are shaded according to conservation and similarity. Residues indicated white on black are identical residues conserved in more than 50% of the sequences. Residues indicated white on grey are similar residues conserved in more than 50% of the sequences. The red boxes indicate the three conserved Walker A (P-loop), Walker A′, and Walker B motifs, which are implicated in nucleotide binding and hydrolysis [84], [85]. The green boxes indicate two conserved basic residues (R189, R218 according to Soj from *B. subtilis*) important for non-specific DNA binding of ParA (Soj) from *B. subtilis* [26] and *C. crescentus* [24].
(EPS)

Figure S4 Phylogenetic tree with the ParB protein (MXAN7476) from *M. xanthus* and selected ParB proteins. As in Figure S1 the light grey shaded proteins derived from plasmids and non-primary chromosomes and the dark grey shaded ParB proteins derived from primary chromosomes. The tree was generated as described for ParA in Figure S1 except that the core region of ParB proteins corresponding to residues 35–293 of the *M. xanthus* ParB was used. The selected sequences are the following (accession no. from top to bottom): NP_233493 (ParBII Vcho), YP_001711991 (ParB pVM01), P07674 (KorB pRK2), NP_422546 (ParB Ccre), NP_628073 (ParB Scoe), YP_001289879 (ParB Mytu), NP_602286 (ParB Cglu), NP_39197s (Spo0J/ParB Bsub), NP_293738 (ParBI Deira), NP_051545 (ParBIII Deira), NP_285326 (ParBII Deira), YP_635579 (ParB Mxan), NP_232398 (ParBI Vcho), NP_742171 (ParB Pput).
(EPS)

Figure S5 Sequence identities of the ParB protein (MXAN7476) from *M. xanthus* and selected ParB proteins. The sequence identity score table was generated as described for ParA in Figure S2.
(EPS)

Figure S6 Alignment of the ParB protein (MXAN7476) from *M. xanthus* and selected ParB proteins. Sequences are the same as in Figure S4. The alignment was made as described for ParA in Figure S3. The red boxes indicate conserved regions within ParB proteins which have been described as boxes I and II and regions 1–4 [22], [45]. The green box in region 3 indicates a conserved arginine residue outside the proposed helix-turn-helix motif (HTH, helices are marked by red rectangles above the alignment) which has been shown to be important for DNA-binding in Spo0J and KorB [43], [44]. The C-terminal part of some ParB proteins around the conserved region 4 has been described as the main dimerization domain [22], [86], [87]. However, also the N-terminal and central DNA-binding domains have been shown to dimerize [88]. Region 1 contains a conserved region around a basic residue (circled in cyan; K7 in SpoOJ of *B. subtilis*), which has been suggested to be involved in activation of nucleotide hydrolysis in the respective ParA partner protein [25].
(EPS)

Figure S7 Purification of His_6_-ParB. SDS-PAGE analysis of purified His_6_-ParB after staining with Coomassie Brilliant Blue R-250. His_6_-ParB with its calculated molecular mass as well as positions of molecular markers are indicated.
(EPS)

Table S1 Primers used in this work.
(DOCX)
