# Supplementary material for: Tracking of Chromosome and Replisome Dynamics in Myxococcus xanthus Reveals a Novel Chromosome Arrangement
Source: PLoS Genet. 2013 Sep 19;9(9):e1003802. doi: 10.1371/journal.pgen.1003802 (PMC3778016; doi:10.1371/journal.pgen.1003802)
Supplement: Table S1 — Primers used in this work. (DOCX) [file pgen.1003802.s008.docx]

**Table S1.** Primers used in this work

| **Primer** | **Sequence^a^** |
| --- | --- |
| DB01 | **GCCGGATCC**CGTGGTGAAAGCAGACATGCAG |
| DB02 | **ATGGCCCTCGAG**CTACTACTCCTTCCTGAGAAGCTTCAA |
| KA436 | **ccgGAATTC**GAGGCGTGTCATCGTCACCCG |
| KA437 | **gcgGGATCCGGCGGAGCC**AGCCACGCGCCTGCGAGG |
| mCherry fwd | **atcGGATCC**ATGGTGAGCAAGGGCGAGGAG |
| mCherry rev | **gccAAGCTT**TTACTTGTACAGCTCGTCCAT |
| cuoA prom | **GGAATTCCAT**ATGCTCTTCACGAATGGATG |
| cuoA+2 | **CCGGAATTC**GCTGCTTCAACGGCATTC |
| AH81 | **ggaattcCAT**ATGGTGAAAGCAGACATGCAGAAGC |
| AH82 | **gcTCTAGA**TCACTTGTACAGCTCGTCCATGCC |
| Mxan5499R | **GCTAGCTAGC**GGCGCGGCGAAGGC |
| Mxan5499F | **CCGAAGCTT**GCGGCTGGCCCAGCAC |
| Mxan4000R | **GCTAGCTAGC**CGCACCGTCCCGTGG |
| Mxan4000F | **CCGAAGCTT**CGTGCACGCCGTCGG |
| Mxan3779R | **GCTAGCTAGC**GTCGAGTTCCTCCTC |
| Mxan3779F | **CCGAAGCTT**CTTCGAGCTGGGAG |
| Mxan1968R | **GCTAGCT**AGCGCGCCCTGGCGCGG |
| Mxan1968F | **CCGAAGC**TTCACACGGGCATCTTG |
| Mxan0733R | **ATCGCTAGCCTGCA**GGTCGTCGTCCACCACCAGC |
| Mxan0733F | **CGAAGCTT**ACGGCATCATCGGCTACTG |
| tetR-YFP-F | **GGAATTCCAT**ATGGTGTCTAGATTAGATAAAAG |
| tetR-YFP-R | **GCCAAGCTT**TTACTTGTACAGCTCGTC |
| EB7 | **CGGGGATCC**GCCCGGCTGACGCCAGC |
| EB8 | **CGGAATTC**AAGCCGCTGCCGCAGTAGAGAC |
| EB11 | **CGTCTAGA**GTGAGCAAGGGCGAGGAG |
| EB12 | **CGCGGATCC**CTACTTGTACAGCTCGTCCATGC |
| EB5 | **GCCAAGCTT**AAGACGCCGGAGGGCTTC |
| EB6 | **GCTCTAGAAATTCGCCAGAACCAGCAGCGGAGCCAGCC**GAAGGGGATGTCGTCGTCG |
| AH46 | **gctctaga**GTGAAAGCAGACATGCAG |
| AH47 | **cgggatcc**CTACTCCTTCCTGAGAAGC |
| AH13 | **CCGGAATTC**GTCAACCAGGACAACCGC |
| AH14 | **GTCGAGGTC**CATGTCTGCTTTCACCACG |
| AH15 | **GCAGACATG**GACCTCGACCGGCTCTTG |
| AH16 | **GCGGGATCC**GAGCGCCAGCAGTGAAGC |
| KA456 | **gcgGGATCC**CGTGCACTGCATCACGCGC |
| KA457 | **gccAAGCTT**TCATCAAGCCACGCGCCTGCG |

^a^ Primer sequences that are not complementary to the template are indicated in bold.
